# Influence of Ligand Functionalization of UiO-66-Based Metal-Organic Frameworks When Used as Sorbents in Dispersive Solid-Phase Analytical Microextraction for Different Aqueous Organic Pollutants

**DOI:** 10.3390/molecules23112869

**Published:** 2018-11-03

**Authors:** Iván Taima-Mancera, Priscilla Rocío-Bautista, Jorge Pasán, Juan H. Ayala, Catalina Ruiz-Pérez, Ana M. Afonso, Ana B. Lago, Verónica Pino

**Affiliations:** 1 Departament of Chemistry (Analytical Division), University of La Laguna, 38206 Tenerife, Spain; ivan.taima.13@ull.edu.es (I.T.-M.); procio@ull.edu.es (P.R.-B.); jayala@ull.edu.es (J.H.A.); aafonso@ull.edu.es (A.M.A.); 2 X-ray and Molecular Materials Lab (MATMOL), Physics Department, University of La Laguna, 38206 Tenerife, Spain; jpasang@ull.edu.es (J.P.); caruiz@ull.edu.es (C.R.-P.)

**Keywords:** metal-organic frameworks, dispersive solid-phase extraction, organic pollutants, analyte partitioning

## Abstract

Four metal-organic frameworks (MOFs), specifically UiO-66, UiO-66-NH_2_, UiO-66-NO_2_, and MIL-53(Al), were synthesized, characterized, and used as sorbents in a dispersive micro-solid phase extraction (D-µSPE) method for the determination of nine pollutants of different nature, including drugs, phenols, polycyclic aromatic hydrocarbons, and personal care products in environmental waters. The D-µSPE method, using these MOFs as sorbents and in combination with high-performance liquid chromatography (HPLC) and diode-array detection (DAD), was optimized. The optimization study pointed out to UiO-66-NO_2_ as the best MOF to use in the multi-component determination. Furthermore, the utilization of isoreticular MOFs based on UiO-66 with the same topology but different functional groups, and MIL-53(Al) to compare with, allowed us for the first time to evaluate the influence of such functionalization of the ligand with regards to the efficiency of the D-µSPE-HPLC-DAD method. Optimum conditions included: 20 mg of UiO-66-NO_2_ MOF in 20 mL of the aqueous sample, 3 min of agitation by vortex and 5 min of centrifugation, followed by the use of only 500 µL of acetonitrile as desorption solvent (once the MOF containing analytes was separated), 5 min of vortex and 5 min of centrifugation. The validation of the D-µSPE-HPLC-DAD method showed limits of detection down to 1.5 ng·L^−1^, average relative recoveries of 107% for a spiked level of 1.50 µg·L^−1^, and inter-day precision values with relative standard deviations lower than 14%, for the group of pollutants considered.

## 1. Introduction

Micro- and mesoporous materials are widespread used for separation and purification purposes due to their excellent adsorption properties. Among these porous materials, metal-organic frameworks (MOFs) have received much attention in the last years due to their unique properties: ordered porous structures, the highest surface areas known and even the possibility of tuning their physiochemical behavior [1]. MOFs are three dimensional porous hybrid materials composed by two main building blocks, metal ions as nodes or connectors and organic molecules as linkers, and the combination of these building units offers a limitless number of possible structures [2]. Thus, MOFs have been included as fashion materials in a wide variety of applications, such as gas storage, catalysis, drug delivery or luminescence sensing, among others [3]. Even so, a great number of concerns still exist surrounding stability and synthesis of MOFs [4], despite the fact that nowadays many MOFs have shown excellent stability under harsh conditions [5] and were able to be synthesized on large scales [6].

The use of MOFs as advanced porous materials for more effective and efficient capture of pollutants from different environmental media is increasing in recent years [7]. An important feature of MOFs, which offers a method to satisfy the adsorbent selection criteria, is the modular nature of the organic linker. The design and modification of MOFs at the molecular level can be achieved generally functionalizing the pore surface. This concept of precise control at the molecular level is probably the most distinguishing characteristic of MOFs as compared to other sorbent materials because they can offer additional adsorption sites and also improve the selectivity of pristine MOFs [8].

The well-known MOF UiO-66 (UiO = University of Oslo (Oslo, Norway)) [9] exhibits exceptional thermal and chemical stability in water and organic solvents, while presenting good adsorption properties [10]. Several studies have already shown the feasibility of functionalizing the UiO-66 material without losing the physicochemical properties of the parent framework, and mainly their advantages in gas capture and gas separation have been analyzed [11]. UiO-66 has shown promising adsorption capacities for organic contaminants such as organic dyes [12] and for inorganic pollutants such as heavy metals [13]. However, the adsorption behavior of emerging pollutants, such as pharmaceutical and personal care products on UiO-66 has been scarcely studied [14], and to the best of our knowledge, there are not studies dealing with functionalized UiO-66 materials in microextraction.

Investigations of MOFs in analytical chemistry are rising [15,16,17,18]. Thus, Zhou et al. reported the first example of MOFs used in analytical chemistry [19], using them in an on-line solid phase extraction (SPE) method. Since then, a variety of MOFs coming from the most widely known families have been tested so far in a number of analytical SPE applications [15,16,18,20] and even in chromatography [17]. The dispersive mode of the miniaturized solid-phase extraction method (D-μSPE) is a successful approach widely used in sample preparation given its simplicity [21]. It requires a strong dispersion of the sorbent (in an amount lower than 500 mg) into an aqueous sample containing analytes (i.e., with the aid of vortex or ultrasounds), followed by proper separation of the sorbent containing extracted analytes from the sample, and further elution/desorption of trapped analytes before the chromatographic determination [16,21,22].

It is important mentioning that, while existing an increasing number of studies with MOFs as sorbents in D-μSPE [16], few authors have paid close attention to study the nature of the interactions established between the MOF sorbent and the contaminants. Indeed, the type of interactions that take place during the extraction has not been established completely [14,23,24]. Thus, Rocío-Bautista et al. evaluated the partitioning of target compounds to different MOFs in D-μSPE [24]. The study highlighted the complexity in achieving adequate predictions for the microextraction performance, with successful results mainly linked to the pore environment, pore size, and pore aperture widths of the MOF, together with a clear influence of the metal nature. The nature of the metal of the MOF and its influence in extraction studies have also been quite recently pointed out by Lirio et al. [14], with high importance given to the radius of the metal.

In this sense, the present study evaluates the analytical performance in D-µSPE of UiO-66 and its derivatives UiO-66-NO_2_ and UiO-66-NH_2_: with the organic linker functionalized with nitro and amino groups, respectively, but maintaining the isoreticular network. The amino (-NH_2_) and nitro (-NO_2_) functional groups were chosen to be representative of polar and hydrophilic functionalities. This linker functionalization produces changes in the physicochemical properties of the frameworks with the purpose of establishing stronger host-guest interactions [25], but it also implies a decrease in surface areas and pore volumes [26]. The MOF MIL-53(Al) is used in this study as a comparative material. The application is devoted to the determination of several water contaminants (of quite different nature) using high-performance liquid chromatography (HPLC) and diode-array detection (DAD), intending a multi-component determination while trying to give insights on the influence of the ligand functionality on the possible MOF-target contaminant interactions favoring the entire D-µSPE-HPLC-DAD method.

## 2. Experimental

### 2.1. Chemicals, Reagents and Materials

Nine analytes of different nature were studied. Carbamazepine (Cbz, 99.0%), 4-cumylphenol (CuP, 99%), 4-*tert*-octylphenol (*t*-OP, 97%), 4-octylphenol (OP, 99%), benzophenone-3 (BP-3, 99.5%) and chrysene (Chy, 98%) were obtained from Sigma-Aldrich (Steinheim, Germany); and progesterone (Pg, >99.99%) was purchased from US Pharmacopeia Reference Standards (Basel, Switzerland). All these compounds were obtained as solid products. A standard solution containing these compounds was prepared in acetonitrile (ACN) Chromasolv^TM^ liquid chromatography (LC) grade, by Honeywell (Seelze, Germany), at a concentration of 100 mg·L^−1^, and stored at 4 °C. Indeno(1,2,3-cd)pyrene (Ind) and triclosan (Tr) were purchased individually as standard solutions, with a concentration of 10 mg·L^−1^ in acetonitrile (ACN) by Dr. Ehrenstorfer GmbH (Augsburg, Germany). Working standard solutions were prepared by dilution in ultrapure water of these standard solutions, with concentrations dependent on the specific experiment. Table S1 of the Electronic Supplementary Material (ESM) shows several characteristics and the structures of the analytes studied.

Ultrapure water (Milli-Q, ultrapure grade) was obtained by a water purification system A10 MilliPore (Watford, UK). Methanol Chromasolv^®^ (LC grade) was purchased from Sigma-Aldrich. HPLC mobile phases were prepared with ultrapure water and ACN Chromasolv^TM^ LC-MS grade. Both phases were filtered with Durapore^®^ membrane filters of 0.45 µm, supplied by Sigma-Aldrich.

0.2 µm polyvinylidene fluoride (PVDF) syringe filters Whatman^TM^ were purchased from GE Healthcare (Buckinghamshire, UK), and used to filter all eluates and standards before HPLC injection.

Pyrex^®^ centrifuge tubes (Corning Inc., Staffordshire, UK) were used in the microextraction procedure, with dimensions of 10 × 2.6 cm and a volume of 25 mL.

Parr Instrument Company (Moline, IL, USA) supplied Teflon solvothermal reactors and stainless steel autoclaves, which were used in the synthesis of the MOFs.

Zirconium chloride (ZrCl_4_, 98%), aluminum(III) nitrate nonahydrate (Al(NO_3_)_3_·9H_2_O, >99.99%), HCl (37%, *v*/*v*), 1,4-benzenedicarboxylic acid (H_2_BDC, 98%), 2-amino-1,4-benzenedicarboxylic acid (NH_2_–H_2_BDC, 99%) and 2-nitro-1,4-dicarboxylic acid (NO_2_–H_2_BDC, ≥99%) were purchased from Sigma-Aldrich and used in the synthesis of MOFs. The solvents used in the synthesis and washing of MOFs include: dimethylformamide (DMF, ≥99.5%), acquired to Merck KGaA (Darmstadt, Germany), and methanol (≥99.8%) purchased from PanReac AppliChem (Barcelona, Spain).

Tap water was taken at the laboratory. Two wastewater samples were supplied by an environmental monitoring laboratory. They were sampled in different areas of Tenerife Island (Canary Islands, Spain) using amber glass recipients properly cleaned, avoiding the formation of bubbles during sampling. They were kept in fridge until reaching the laboratory, and then they were filtered through 0.45 μm filters and kept in the dark at 4 °C until analysis.

### 2.2. Synthesis of MOFs

The MOFs used in this study were synthesized according to the procedure reported by Katz et al. [27]. A standard upscale synthesis of UiO-66 was performed by dissolving 233 mg of ZrCl_4_ (1 mmol) and 246 mg of H_2_BDC (1.5 mmol) in 15 mL of DMF and 1 mL of concentrated HCl. The resulting mixture was heated in a solvothermal reactor at 150 °C for 24 h. After the solution was cooled to room temperature, the resulting solid was filtered and repeatedly washed with DMF, methanol, and heated at 150 °C for 24 h in order to remove guest molecules from the pores of the crystalline structure. UiO-66-NH_2_ and UiO-66-NO_2_ MOFs were synthesized analogously by replacing H_2_BDC with the equivalent molar amounts of NH_2_–H_2_BDC and NO_2_–H_2_BDC, respectively.

MIL-53(Al) was prepared according to Loiseau et al. [28]. Briefly, 288 mg of H_2_BDC (1.7 mmol) and 1.3 g of Al(NO_3_)_3_·9H_2_O (3.5 mmol) were mixed in a solvothermal reactor using 15 mL of ultrapure water. The solution was then heated at 220 °C for 3 days. Afterwards, the autoclave was cooled down to room temperature and the obtained white product was isolated by filtration, washed with water, and air-dried at 50 °C. The MOF was finally heated at 400 °C during 16 h for the activation.

All synthetic conditions and obtained yields for the MOFs are summarized in Table 1.

### 2.3. Instruments and Equipment

The HPLC used in the determination was a 1260 Infinity model purchased from Agilent Technologies (Santa Clara, CA, USA), in combination with a DAD 1260 Infinity model also from Agilent Technologies. The quantification wavelengths of the DAD were set at 240 nm for Pg, 254 nm for Ind, 270 nm for Chy, 280 nm for CuP, *t*-OP and OP, and 289 nm for Cbz, BP-3 and Tr. The HPLC system includes a Rheodyne injection valve with an injection loop of 20 μL, supplied by Supelco (Bellefonte, PA, USA). The separation of target analytes was carried out in an ACE Ultra Core 5 SuperC18 (5 μm, 150 × 4.6 mm) analytical column, obtained from Symta (Madrid, Spain), with a safeguard column Pelliguard LC-18 purchased to Supelco. ACN and ultrapure water were employed as mobile phases using a linear gradient at a constant flow rate of 1 mL·min^−1^. The chromatographic method starts at 50% (*v*/*v*) of ACN, keeping it constant for 5 min, then increased up to 80% (*v*/*v*) in 2 min, then increased up to 83% (*v*/*v*) in the next 2.5 min, and finally reaching 100% (*v*/*v*) of ACN in the next 3.5 min.

A vortexer from Reax-Control Heidolph^TM^ GmbH (Schwabach, Germany) and a centrifuge model 5720 Eppendorf^TM^ (Eppendorf, Hamburg, Germany) were utilized in the D-µSPE procedure.

The synthesis of the MOFs was carried out in a Universal model UF30 oven supplied by Memmert (Schwabach, Germany).

Phase identification of all MOFs was carried out by X-ray powder diffraction. A X’Pert Diffractometer supplied by PANalytical (Eindhoven, The Netherlands) and operating with Bragg-Brentano geometry was used. Data collection was carried out using Cu K_1_ radiation (λ = 1.5418 Å) over the angular range from 5.01° to 80.00° (0.02° steps) with a total exposure time of 30 min.

An Affinity-1 Fourier transform-infrared (FTIR) spectroscope from Shimadzu (Kyoto, Japan) was used in the identification of the functional groups incorporated to UiO-66.

The nitrogen adsorption isotherms were measured on a Gemini V2365 Model, supplied by Micromeritics (Norcross, GA, USA), surface area analyzer at 77 K in the range 0.02 ≤ P/P_0_ ≤ 1.00. The Brunauer, Emmet and Teller (BET) method was used to calculate the surface area.

Particle sizes of the crystals were determined at 25 °C by dynamic light scattering (DLS) using the Zetasizer equipment from Malvern Instruments (Malvern, UK), with the Zetasizer software v. 7.03.

### 2.4. Dispersive Miniaturized Solid-Phase Extraction Procedure (D-µSPE)

All conditions of the D-µSPE method using all studied MOFs as sorbents were optimized, including the conditions of both: the extraction and the desorption steps. Under optimum conditions, the extraction is carried out adding 20 mg of UiO-66-NO_2_ over 20 mL of water sample (or aqueous standard, depending on the experiment) in a 25 mL Pyrex^®^ centrifuge tube. The tube is then subjected to vortex stirring for 3 min, to increase the strength of the interaction between the sorbent and the analytes. Then, the phases are separated by centrifugation (1921× *g* during 5 min) and the supernatant aqueous phase is carefully removed. For the desorption step, 500 µL of ACN are added to the UiO-66-NO_2_ left in the tube containing extracted analytes. Vortexing is applied for another 5 min followed by centrifugation during 5 min at 1921× *g*. The eluate is filtered through 0.2 µm PVDF syringe filters before being injected in the HPLC.

## 3. Results and Discussion

### 3.1. Chromatographic Method

Contaminants selected in this study include polycyclic aromatic hydrocarbons, drugs, phenols, and personal care products, with the purpose of having a variety of quite different analytes, thus covering different possible interaction mechanisms with the MOFs. The determination of the nine target compounds was carried out using HPLC-DAD, employing proper quantification wavelengths for each analyte. The optimum conditions for the separation were summarized in Section 2.3, with the overall separation requiring less than 13 min, as it can be observed in Figure S1 of the ESM.

Several quality analytical parameters of the calibrations obtained by HPLC-DAD are shown in Table S2 of the ESM. Calibration curves present adequate linearity, with correlation coefficient (R) values higher than 0.9983. The limits of detection (LOD) and the limits of quantification (LOQ) were calculated as the signal to noise (S/N) ratio of 3 and 10, respectively. LOD and LOQ values were verified by preparation of standards at such levels of concentration. LODs varied from 0.02 μg·L^−1^ for Chy to 1.00 μg·L^−1^ for CuP. The precision of the chromatographic method was calculated using three standards at concentration levels not utilized in the calibration curve (but included within the calibration range): 6 μg·L^−1^, 30 μg·L^−1^ and 70 μg·L^−1^ (*n* = 5). The obtained results are included in Table S3 of the ESM. In all cases, relative standard deviations (RSD, in %) values were lower than 3.7% for the lowest concentration level tested and 2.9% for the highest concentration level injected. Regarding the precision of the chromatographic retention times, RSD values were always lower than 0.21% (*n* = 15).

### 3.2. Synthesis and Characterization of Studied MOFs

Following an isoreticular synthesis, a family of MOFs based on the UiO-66 structure was obtained from the two different linker ligands: NH_2_−H_2_BDC and NO_2_−H_2_BDC [25]. The crystal and particle size of the UiO-66 and UiO-66-X (X = NH_2_ and NO_2_) materials were controlled by modulated synthesis with HCl (as detailed in Section 2.2 and Table 1). All compounds were obtained in high yields without loss of crystallinity or porosity by including HCl in the reaction mixtures during the synthesis.

The X-ray diffraction patterns obtained for the as-synthesized samples (see Figures S2 and S3 of the ESM) revealed that the materials are crystalline and the two functionalized compounds are isostructural with the parent material UiO-66, which demonstrates that the tagged UiO-66-X MOFs are topologically equivalent with UiO-66. It is important to proof this equivalence in order to achieve proper comparison when using these materials as sorbents, to clearly link results to the organic ligand nature.

N_2_ adsorption/desorption isotherms were collected at 77 K (Figure S4 of the ESM) and the Brunauer, Emmett and Teller (BET) surface areas were calculated and all the materials were found to retain porosity. The BET surface area data was found to decrease in surface area with the functionalization of the pores, from ∼1342 m^2^·g^−1^ in the parent UiO-66 to ~794 m^2^·g^−1^ for UiO-66-NH_2_ and ∼771 m^2^·g^−1^ for UiO-66-NO_2_, and they are in agreement with previous reported values [27].

The presence of the functional groups on the linkers was further evidenced by characterizing the MOFs with FTIR spectroscopy (Figure S5 of the ESM). Thus, UiO-66-NH_2_ displays a broad absorption band at 3336 cm^−1^ that is assigned to the N-H stretching modes. A band at 1546 cm^−1^ and a band at 1389 cm^−1^ are attributed respectively, to the asymmetric (ν(NO)_asym_) and symmetric (ν(NO)_sym_) stretching modes of the nitro group in UiO-66-NO_2_.

MIL-53(Al) was used in this study for comparative purposes, because it has previously demonstrated its successful performance as sorbent in D-µSPE. The diffraction pattern (Figure S3 of the ESM) shows that the synthesized compound presents the crystal structure of MIL-53(Al) and the calculated Brunauer, Emmett and Teller (BET) surface area is in agreement with the reported value [24].

The particle size distribution of the studied MOFs, shown in Figure S6 of the ESM, shows certain dispersion, particularly for MIL-53(Al)—from 0.1 to 1.5 µm—and for UiO-66—from 0.1 to 1.1 µm, with narrower distribution for UiO-66-NO_2_ and UiO-66-NH_2_. In general, most crystals have particles sizes ranging between ~0.4–0.5 µm and ~0.7–0.8 µm, thus showing quite similar values.

### 3.3. Screening of MOFs as Sorbents in D-µSPE-HPLC-DAD

D-µSPE was selected as microextraction approach in this study given its simplicity and high analytical performance [16]. An initial screening study was carried out in order to study the microextraction performance of UiO-66, UiO-66-NH_2_ and UiO-66-NO_2_ in the D-µSPE of target contaminants. The main difference among these MOFs, as mentioned above in the characterization study, is the nature of the organic ligand, having all the same topology and quite similar surface areas. In this screening study, the MOF MIL-53(Al) was also included in the comparison for having been pointed out as adequate sorbent in common microextraction applications of similar analytes [24].

The D-µSPE method was initially performed with common extraction conditions. Thus, low amounts of MOF were used to fulfil microextraction requirements, minimization of costs, and proper environmental goals. The analytes, once trapped by the MOF, were desorbed using ACN as elution solvent, for being compatible with the HPLC mobile phases used. In this sense, the initial working conditions included 20 mg of MOF, 20 mL of an aqueous standard (containing all analytes at a concentration level of 5.00 µg·L^−1^), 5 min of vortex agitation, and 5 min of centrifugation (at 1921× *g*). Afterwards, the supernatant was discarded and 0.500 mL (to avoid losses of preconcentration during the process) of ACN were added as elution solvent, followed by 5 min of vortex and 5 min of centrifugation (at 1921× *g*).

Results obtained under these conditions revealed that UiO-66-NO_2_ presented adequate extraction efficiencies for many analytes, and that in practically all cases (only excluding CuP), MIL-53(Al) was not the best MOF to be chosen as sorbent despite previous studies [24]. Therefore, we used the information obtained from this screening study to optimize the entire D-µSPE-HPLC-DAD method using UiO-66-NO_2_ as sorbent.

### 3.4. Optimization of the D-µSPE-HPLC-DAD Method Using UiO-66-NO_2_

The D-µSPE-HPLC-DAD method with UiO-66-NO_2_ was optimized having as targets the maximization of the extraction efficiency for the highest number of possible of contaminants, and the minimization of the amount of MOF and solvents in the procedure. Main variables studied in the one-factor-at a time optimization of the method were: amount of MOF sorbent, extraction and elution times, number of elution steps and nature of the elution solvent.

**Figure 1:**
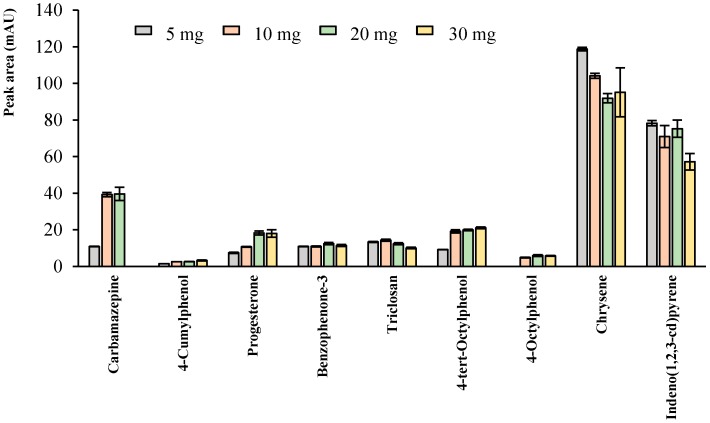
Effect of the amount of UiO-66-NO_2_ on the extraction efficiency for all analytes in D-µSPE-HPLC-DAD. Specific conditions are described in Section 3.4. Experiments were carried out in triplicate.

The first variable optimized was the amount of UiO-66-NO_2_, with tested values ranging between 10 and 30 mg. These studies were carried out with aqueous standards (10.0 µg·L^−1^ of Cbz, Tr and *t*-OP, and 2.50 µg·L^−1^ for the rest of analytes), and the remaining conditions already fixed during the screening (Section 3.3). Figure 1 shows the results obtained monitoring the extraction efficiencies in terms of peak areas. In general, best results were obtained when using 10 or 20 mg of UiO-66-NO_2_, without significant differences in the performance among them, except for Pg, which achieved much better results when using 20 mg. For this reason, 20 mg was selected as the optimum amount of UiO-66-NO_2_.

The second variable considered in this optimization was the extraction time, with the extraction step assisted by vortex, utilizing times between 1 and 5 min. Vortex times higher than 5 min are not advisable (unhealthy) and thus were not tested. These experiments were performed using 20 mg of MOF, and the fixed conditions above mentioned. Figure S7 of the ESM shows the obtained results, which clearly point out to 3 min as the optimum value.

The third variable included in the optimization was the elution time, also assisted by vortex, with times also varying between 1 and 5 min, using as fixed conditions: 20 mg of MOF, 3 min for the vortex-assisted extraction time, and the remaining conditions fixed as in previous experiments. Figure S8 of the ESM shows clear improvements in the elution with longer times, and thus 5 min was selected as the optimum.

**Figure 2:**
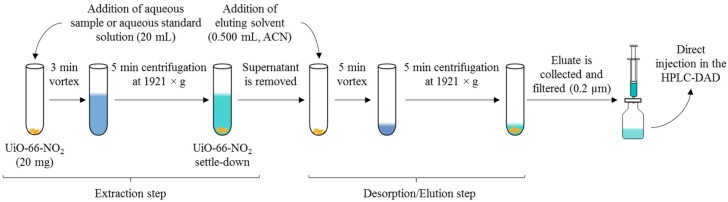
Scheme of the entire D-µSPE-HPLC-DAD method using the MOF UiO-66-NO_2_ under optimum conditions.

The elution step in D-µSPE with MOFs has been pointed out as the critical step to achieve adequate analytical performance [29]. Therefore, the number of elution steps and the nature of the elution solvent were also considered in this optimization. Regarding the number of elution steps, it was evaluated the use of two elution steps (each one with 0.250 mL of ACN) versus the use of one single elution step (0.500 mL of ACN). It can be observed from Figure S9 of the ESM the absence of significant improvements when increasing the number of elution steps. Therefore, one single elution step was preferred. This also permits a decrease in the overall analysis time, which is advisable. Regarding the nature of the elution solvent, different solvents (compatible with HPLC mobile phases) were compared: ACN, methanol, and acetone; using all already optimized conditions of the method. Results (included in Figure S10 of the ESM) point out the adequacy of ACN. A summary of the entire optimized D-µSPE-HPLC-DAD method is included in Figure 2.

### 3.5. Influence of the UiO-66 Ligand Functionalization in the Overall Efficiency of the D-µSPE-HPLC-DAD Method

**Figure 3:**
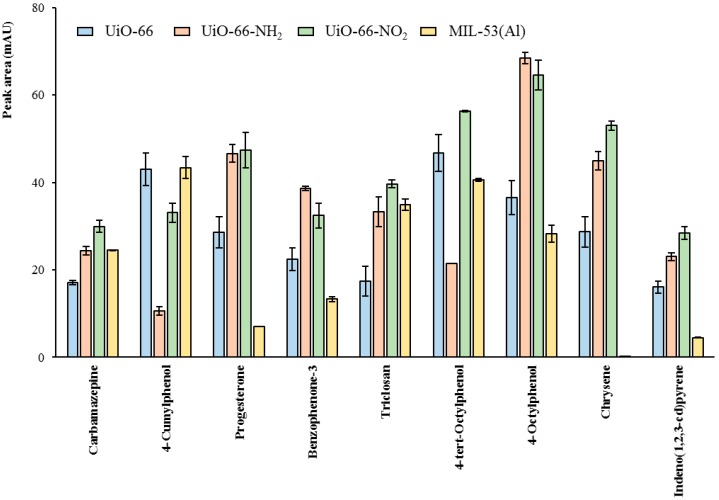
Comparison between the different MOFs used in terms of extraction efficiency with the optimum D-µSPE-HPLC-DAD method. Fixed optimum conditions as described in the text. Experiments were carried out in triplicate.

After completion of the optimization, the D-µSPE-HPLC-DAD method was carried out using UiO-66, UiO-66 derivatives, and MIL-53(Al) as sorbents. The E_R_ values obtained for the target analytes with the different MOFs are included in Figure 3. Results revealed that UiO-66-NO_2_ was the best MOF as sorbent for seven out of the nine contaminants studied, and thus it can be considered a generic sorbent if intending a multi-component determination.

Nevertheless, apart for the importance of utilizing this MOF to set up a multi-component monitoring method through D-µSPE, the obtained results are highly valuable in order to get understanding on the nature of possible interactions MOFs-analytes. However, one should keep in mind that the E_R_ values refer to the complete microextraction method, comprising two steps: the adsorption of the analytes by the MOF extractant and the elution/desorption process. Therefore, the affinity of the MOF for a particular analyte cannot be made exclusively based on the E_R_ value, since this evaluate a complete process in which desorption readiness (weak analyte-MOF interaction) is favored.

In general, the results introduced in this study indicate that the functionalization by means of polar groups is a noteworthy factor affecting positively the total efficiency of the method (understood as extraction/elution process ability) for the studied analytes of quite different nature. This result shows that the reduction in effective pore size by functional groups is not critical in the analytical procedure, since the electronic properties of these groups favor the analyte-MOF interaction. The UiO-66 has a moderate pore window size of around 7 Å in diameter and moderate pore size diameter of 11 Å, whereas the UiO-66-X variants reduce the window opening and pore sizes [30]. This implies that the pore window size is large enough for small molecules, but the bulkier ones may have hindering problems and they most likely interact with the MOF surface.

Two adsorption sites for pollutants molecules can be distinguished in the UiO-66-X family, the Zr_6_O_4_(OH)_4_ cluster, a hydrophilic area where water, acetic acid and other groups can be coordinated in some MOFs [31]; and the ligand environment which changes from the more hydrophobic X = H to the more hydrophilic X = NH_2_ or NO_2_. The adsorption mechanisms may include hydrophobic effects, π–π electron donor–acceptor interactions, electrostatic attractions, [32] or even stronger interactions such as chemical bond or hydrogen bond [33,34].

Analytes accessing the pores window will have a strong interaction with the inside pore walls, which probably improve the extraction step but at the same time, makes more difficult the elution step. The host voids in functionalized materials can act as a “tweezer”, providing suitable electronic environments to trap the guest molecules [8]. The moderate pore size will make the adsorbed molecules have a closer distance and stronger interactions with the inside pore walls, thus making difficult the elution process with organic eluents. Therefore, we cannot conclude that higher real recovery values listed in Figure 3 necessarily imply better analyte-MOF interactions, they just represent the minimum value of the quantity of analyte adsorbed by the MOF (either at the pores or at the surface).

Considering this, the best extraction performance occurs for the 4-octylphenol with both NH_2_- and NO_2_-functionalized UiO-66 with E_R_ values larger than 60%, and in general, UiO-66-NO_2_ seems to be the best extractant material as remarked before. The influence of the narrower pore in the decorated UiO-66 MOFs can be observed in the trend for the three phenols analyzed. They follow a trend where the 4-cumylphenol (CuP) is the bulkier, followed by the 4-*tert*-octylphenol (*t*-OP) and the 4-octylphenol (OP). The E_R_ values for the NH_2_-UiO-66 and NO_2_-UiO-66 follow an inverse trend, OP > *t*-OP > CuP, whilst for the bare UiO-66 and MIL-53(Al) the E_R_ values are similar (around 40%). One can conclude that for bulkier analytes the adsorption is hindered, and the E_R_ is reduced. In the case of the MOF MIL-53(Al), its breathing nature implies that the structure can change to the closed-form upon adsorption of guests, in particular, water or some analytes can be triggering this transformation and the resulting analytical performance is affected.

In general, the functionalized NH_2_- and NO_2_-UiO-66 MOFs outperform the bare UiO-66 in recovery values. This may be due to various reasons: (i) The amino -and nitro- decorations as H-bond donor and acceptor groups increase the anchoring sites for guest molecules, and these electron-withdrawing groups could lead to effective adsorption from charge-transfer interactions between the functionalized groups and these guest molecules [35]; (ii) The more hydrophilic environment in the pore surface caused by the NH_2_ and NO_2_ groups may promote a better elution with the non-polar organic solvents.

In the case of triclosan (Tr), it seems that a larger number of anchoring sites in the form of H-bond acceptors or donors notably increase the extraction ability of the UiO-66 [36]. This situation is also observed in Figure 3, where both NH_2_- and NO_2_-UiO-66 outperform bare UiO-66 in the extraction of triclosan, and a similar trend is observed for carbamazepine (Cbz), progesterone (Pg), and benzophenone-3 (BP-3).

In the case of the PAHs included in this analysis, chrysene (Chy) and indeno(1,2,3-cd)pyrene (Ind), the functionalized UiO-66 MOFs also perform better than the bare one. This situation seems contradictory, since more hydrophobic, bulky analytes are being better recovered by more hydrophilic, narrow pore NH_2_- and NO_2_-UiO-66 materials. This behavior may be explained taking into account the favored elution process of loosely linked PAHs on the surface of the functionalized MOFs. Probably, these bulky PAHs are adsorbed on the surface of the MOFs and the slightly more hydrophilic environment of the functionalized UiO-66 help in the elution process, and we therefore observe an increase in the total E_R_ factor. Also, we cannot discard some competitive interactions of the MOFs with the other analytes that lead to a better recovery of the PAHs in this case.

Clearly, to establish relationships between the effects of the functionalization of MOFs on their sorption capacities results a very difficult task, because there are numerous factors to consider [37]. Among them, pore size reduction and molecular sieving effect [38], changes in polar character or electronic environments of the frameworks, energy effects [39] or other factors (such as intrinsic defects, co-adsorption, or diffusive transport in the material pores, as those most remarkable). Moreover, from the point of view of organic pollutants trapped by these sorbents, the physicochemical parameters such as molecular sizes, shapes, polarities, polarizabilities, interaction abilities, solubility, hydrophilicity, acidity and so on, also need consideration. An approach to the fundamental understanding of mechanisms and interactions is highly required for the design of better materials, even selective, for microextraction processes.

### 3.6. Quality Analytical Parameters of the Optimized D-µSPE-HPLC-DAD Method

The analytical method using D-µSPE-HPLC-DAD and the MOF UiO-66-NO_2_ under the optimized conditions described (Figure 2) was validated. Calibration curves were obtained subjecting aqueous standards to the entire method. Several of the quality analytical parameters obtained are shown in Table 2. Correlation coefficients (R) were higher than 0.9966 in all cases. LODs were calculated as the concentration in the aqueous sample able to generate a signal to noise ratio of three (S/N = 3) after the overall microextraction and chromatographic procedure, and LOQs as ten times signal to noise ratio (S/N = 10). LODs and LOQs were verified by preparation of aqueous standards at those levels. Obtained LODs ranged from 1.5 ng·L^−1^ for Chy and Ind to 300 ng·L^−1^ for CuP and *t*-OP. It is important to highlight the low LODs achieved with the current microextraction method, particularly considering that DAD is used. A comparison with other methods, which also use solid-based dispersion techniques in combination with HPLC-DAD or HPLC-UV, are included in Table S4 of the ESM. Clearly, the current study presents better sensitivity, and it presents the difficulty of dealing with multi-component determination.

The inter-day precision (RSD, in %) of the entire D-µSPE-HPLC-DAD method was studied, by triplicate, at two spiked levels during three non-consecutive days (*n* = 3, intra-day). Table 3 shows that inter-day RSD values ranged from 4.1% for Pg to 14% for Cbz at the lowest spiked level, and from 4.3% for OP to 9.7% for Tr at the highest spiked level. Intra-day RSD values were always lower than 12%.

Average relative recoveries (RR, in %) were of 107% at the lowest spiked level and of 98.0% at the highest spiked level. Real extraction efficiencies (E_R_, in %), for the entire D-µSPE-HPLC-DAD method range from 22.0% for Cbz to 69.6% for OP at the lowest spiked level. Several authors have pointed out the difficulties in reaching E_R_ values close to 100% in any microextraction procedure. Indeed, E_R_ values are generally not reported in microextraction studies (see Table S4 of the ESM). In any case, the validity of any E_R_ value for an analyte in a specific microextraction method is dependent on how sensitive and reproducible is a method for a particular application [16,40].

### 3.7. Analysis of Wastewaters and Tap Water Samples Using the Optimized D-µSPE-HPLC-DAD Method

One tap water and two wastewaters samples were analyzed using the optimized D-µSPE-HPLC-DAD method. As it can be observed in Table S5 of the ESM, none of the nine analytes were detected in these samples.

Wastewater-1 was used as blank matrix to perform precision and recovery studies that serve, in addition, to evaluate the matrix effect given its complexity. The obtained results are also shown in Table S5 of the ESM, using a quite low spiked level of 1.50 µg·L^−1^. From the obtained results, there is a clear matrix effect for analytes such as Cbz, Tr and Pg. For this type of samples, matrix-matched calibrations are recommended.

In any case, it is important to mention that the obtained RSD values (in %) were lower than 17%, which is adequate for this microextraction method considering the low spiked level used for the wastewater sample and its complexity.

## 4. Conclusions

Four MOFs (MIL-53(Al), UiO-66, UiO,66-NH_2_ and UiO-66-NO_2_) were successfully synthesized, characterized and tested as sorbents for a D-µSPE method that implies monitoring of nine pollutants of different nature: carbamazepine, 4-cumylphenol, progesterone, benzophenone-3, triclosan, 4-*tert*-octylphenol, 4-octylphenol, chrysene and indeno(1,2,3-cd)pyrene.

The dispersive method in combination with HPLC-DAD was properly optimized using the UiO-66-NO_2_ MOF, selected as the sorbent which offers highest extraction efficiencies for seven out of the nine analytes, thus being a generic sorbent for this multi-component determination. Low amounts of MOF (20 mg), low sample volumes (20 mL), short sample preparation times (8 min for extraction and 10 min for desorption, both using vortex), and the minimization of the organic solvent needed in the elution step (500 µL) were the optimum conditions for this microextraction method. Limits of detection down to 1.5 ng·L^−1^ were achieved for Chy and Ind despite using DAD, as well as proper analytical performance results such as adequate recovery, extraction efficiency and inter-day precision.

An insight on the possible interactions established between these MOFs and the studied analytes, as a function of the nature of the functionalization of the organic ligand in the MOF, while keeping constant the remaining topological conditions of the crystals, was given. The total efficiency of the D-µSPE-HPLC-DAD method was positively influenced by the presence of functionalization groups in the ligands of UiO-66, particularly due to the polar character given to the organic linkers. Nevertheless, not all results can be justified only based on the polar character of the organic linkers. As main factors, it is important to highlight the pore size of a MOF and the molecular sieving effect, changes in the polar character or the electronic environments of the frameworks, energy effects, as well as intrinsic characteristics of analytes experiencing partitioning to the MOFs.

The results of the current study will serve to better design of MOFs to be used as sorbents in D-µSPE, intending tailored microextractions.

## Figures and Tables

**Table 1:** Synthetic conditions of MOFs and yields obtained.

MOF	Structure (Detailed Functionalization for UiO-66)	Metal (mg)	Ligand (mg)	Solvent (mL)	Modulator/mL	Yield (%)
UiO-66	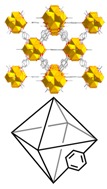	Zr^4+^ (233)	terephthalic acid (246)	DMF (15)	HCl (37%, *v*/*v*)/1	95
UiO-66-NH_2_	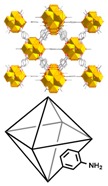	Zr^4+^ (233)	2-aminoterephthalic acid (271)	DMF (15)	HCl (37%, *v*/*v*)/1	78
UiO-66-NO_2_	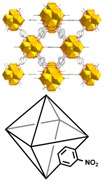	Zr^4+^ (233)	2-nitroterephthalic acid (317)	DMF (15)	HCl (37%, *v*/*v*)/1	97
MIL-53(Al)	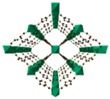	Al^3+^ (1300)	terephthalic acid (288)	H_2_O (15)	-	45

DMF: dimethylformamide.

**Table 2:** Several quality analytical parameters of the D-μSPE-HPLC-DAD method.

Analyte	Calibration Range (μg·L^−1^)	R	s_y/x_^a^	Slope ± SD ^b^	LOD (ng·L^−1^)	LOQ (ng·L^−1^)
Carbamazepine	0.05–5.74	0.9989	0.27	2.3 ± 0.2	5.0	16.7
4-Cumylphenol	0.80–5.74	0.9966	0.11	0.5 ± 0.1	90	300
Progesterone	0.01–5.74	0.9980	0.99	6.5 ± 0.5	2.4	8.00
Benzophenone-3	0.05–5.74	0.9991	0.38	3.8 ± 0.2	4.5	15.0
Triclosan	0.50–5.00	0.9982	0.17	1.3 ± 0.1	30	100
4-*tert*-Octylphenol	0.50–4.00	0.9995	0.09	1.8 ± 0.1	90	300
4-Octylphenol	0.10–5.00	0.9998	0.10	2.4 ± 0.1	15	50.0
Chrysene	0.01–5.74	0.9984	4.8	37 ± 2	1.5	5.00
Indeno(1,2,3-cd)pyrene	0.01–5.74	0.9986	1.6	13 ± 1	1.5	5.00

^a^ standard deviation of the regression (or error of the estimate). ^b^ confidence intervals for the slope (*n* = 6) with a signification level of 95%.

**Table 3:** Analytical performance of the entire D-µSPE-HPLC-DAD method in terms of relative recovery, extraction efficiency, and inter-day precision with aqueous standards.

Analyte	Spiked Level 1 (1.50 µg·L^−1^)				Spiked Level 2 (4.50 µg·L^−1^)			
	E_R_^a^ (%)	RR ^b^ (%)	Inter-Day RSD ^c^ (%)	Intra-Day RSD Range ^d^ (%)	E_R_^a^ (%)	RR ^b^ (%)	Inter-Day RSD ^c^ (%)	Intra-Day RSD Range ^d^ (%)
Cbz	22.0	99.4	14	1.0–12	15.6	100	8.8	6.9–11
CuP	35.2	126	9.3	4.7–8.3	21.1	100	9.6	5.4–6.7
Pg	51.0	111	4.1	3.3–4.4	42.8	88.8	6.7	3.2–4.8
BP-3	29.2	112	9.4	3.2–7.7	25.7	91.9	8.1	2.6–3.6
Tr	40.8	95.0	8.2	5.4–9.5	43.0	104	9.7	5.6–8.9
*t*-OP	53.5	118	7.2	4.1–8.2	45.7	102	5.7	2.5–3.7
OP	69.6	102	7.5	6.1–9.5	63.9	90.5	4.3	1.2–2.4
Chy	39.4	109	5.5	2.0–7.4	43.8	127	8.7	3.3–9.6
Ind	27.1	87.3	9.1	4.1–8.4	24.3	79.2	6.3	2.9–5.9

^a^ extraction efficiency calculated considering the preconcentration achieved with the microextraction method. ^b^ relative recovery. ^c^ relative standard deviation for the inter-day precision (*n* = 9, 3 non-consecutive days). ^d^ intra-day relative standard deviation range (*n* = 3).

## Supplementary Materials

The supplementary materials are available online.
