# Supplementary material for: Influence of Ligand Functionalization of UiO-66-Based Metal-Organic Frameworks When Used as Sorbents in Dispersive Solid-Phase Analytical Microextraction for Different Aqueous Organic Pollutants
Source: Molecules. 2018 Nov 3;23(11):2869. doi: 10.3390/molecules23112869 (PMC6278427; doi:10.3390/molecules23112869)
Supplement: Supplementary file 1 [file molecules-23-02869-s001.pdf]

Supplementary Material

Influence of ligand functionalization of UiO-66-based metal-organic frameworks when used as sorbents in dispersive solid-phase analytical microextraction for different aqueous organic pollutants

Iván Taima-Mancera[1], Priscilla Rocío-Bautista[1], Jorge Pasán[2], Juan H. Ayala[1], Catalina Ruiz-Pérez[2], Ana M. Afonso[1], Ana B. Lago[2,], Verónica Pino[1,*]**

[1]*Departament of Chemistry (Analytical Division), University of La Laguna, Tenerife, 38206 Spain*

[2]*X Ray and Molecular Materials Lab (MATMOL), Physics Department, University of La Laguna, Tenerife, 38206 Spain*

Table of contents

FIGURES

Figure S1 .... page S1

Figure S2 .... page S2

Figure S3 .... page S3

Figure S4 .... page S4

Figure S5 .... page S5

Figure S6 .... page S6

Figure S7 .... page S7

Figure S8 .... page S8

Figure S9 .... page S9

Figure S10 .... page S10

TABLES

Table S1 .... page S11

Table S2 .... page S13

Table S3 .... page S14

Table S4 .... page S15

Table S5 .... page S16

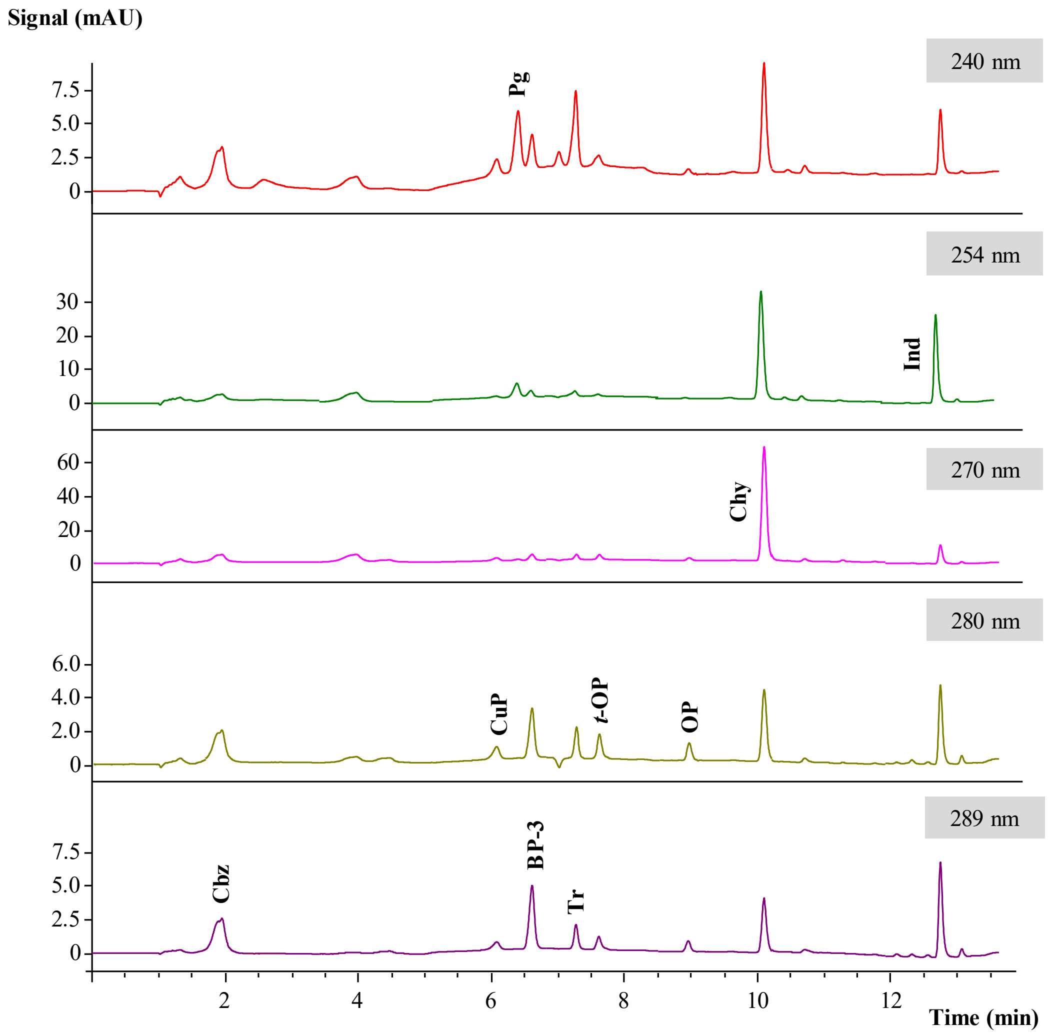

Figure S1. Representative chromatogram of a standard (100 μg·L^{-1}) containing all analytes.

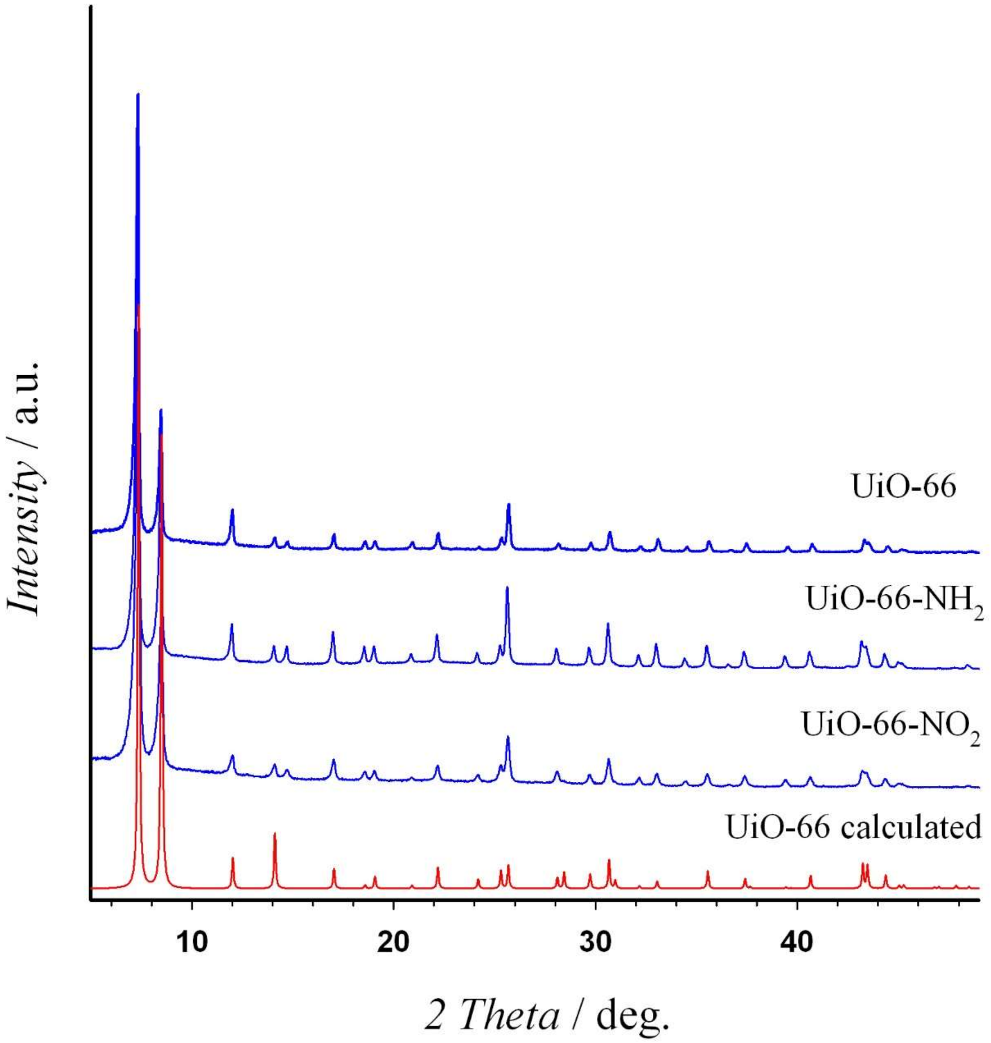

Figure S2. XRD patterns of UiO-66, UiO-66-NO_2 and UiO-66-NH_2 MOFs.

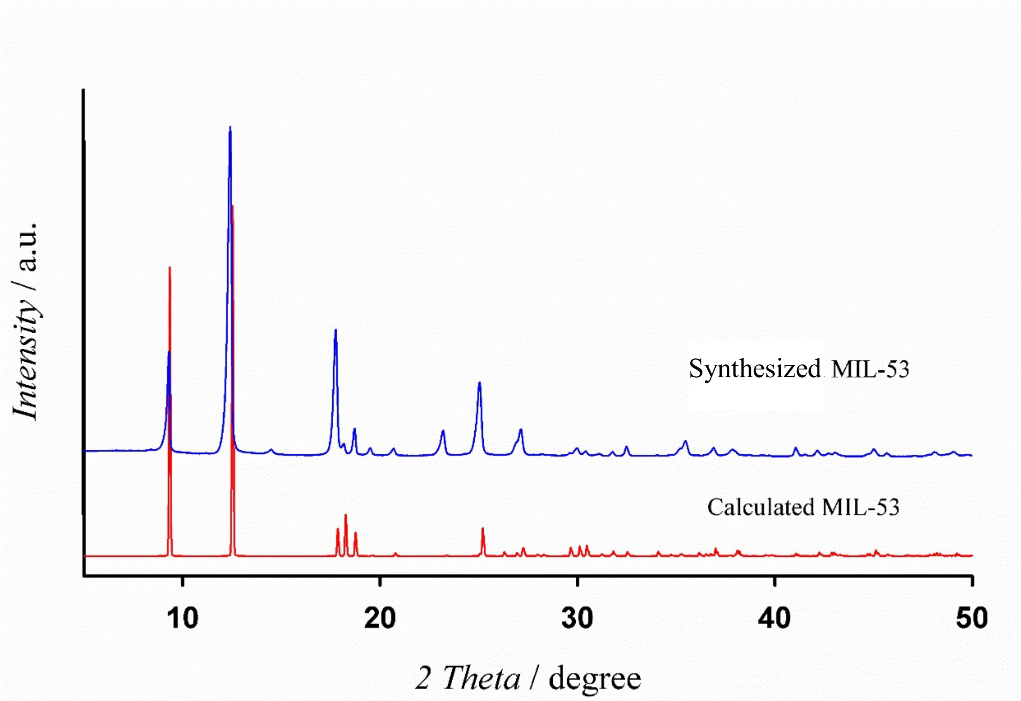

Figure S3. XRD patterns of MIL-53(Al) MOF.

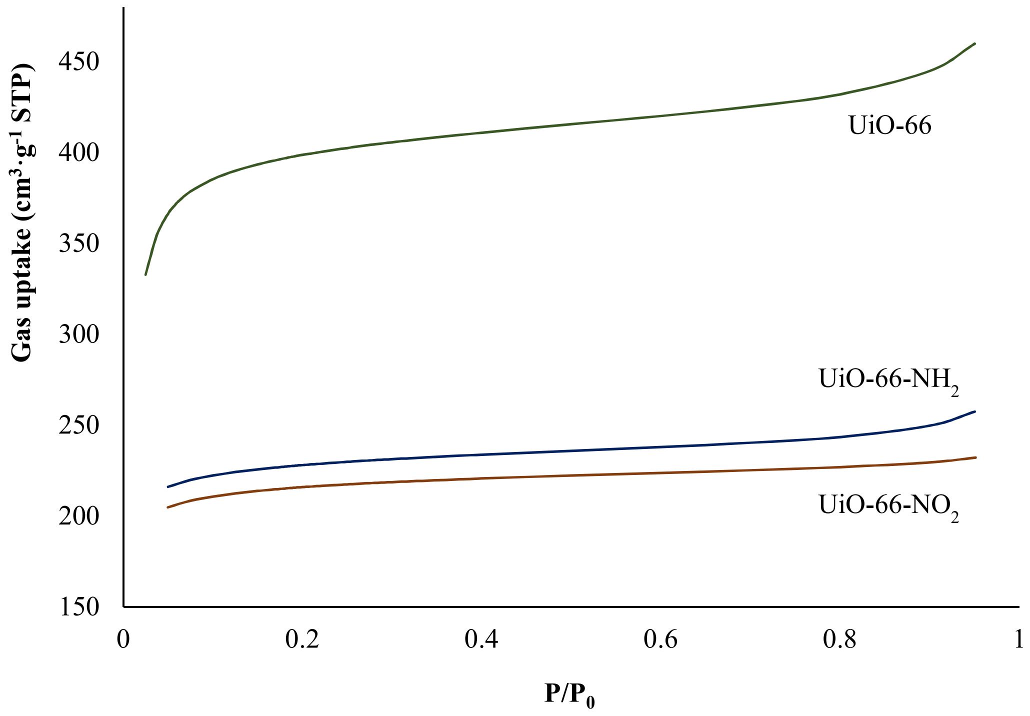

Figure S4. Nitrogen adsorption/desorption isotherms of UiO-66, UiO-66-NH_2 and UiO-66-NO_2 MOFs.

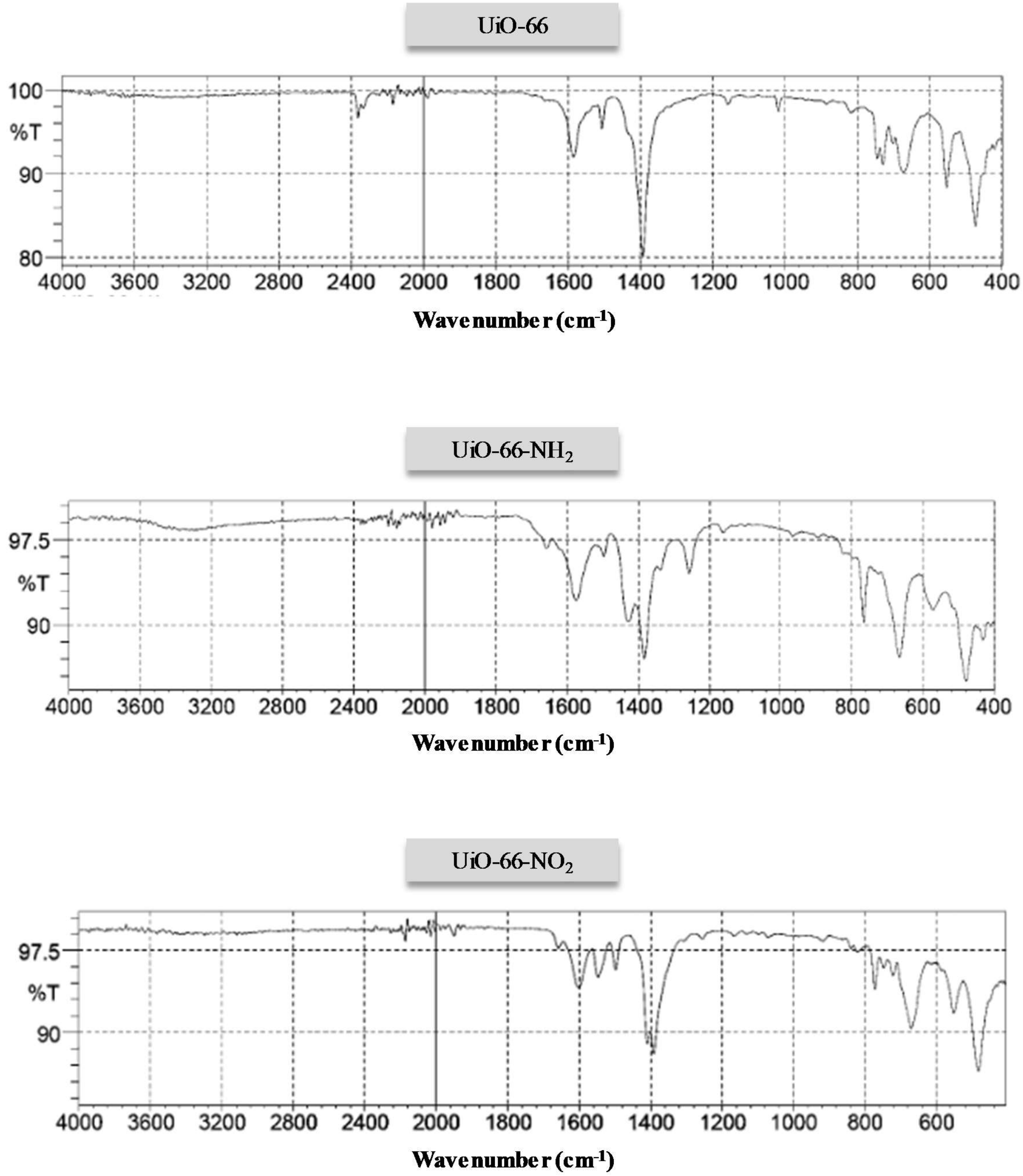

Figure S5. FTIR spectra of synthesized MOFs.

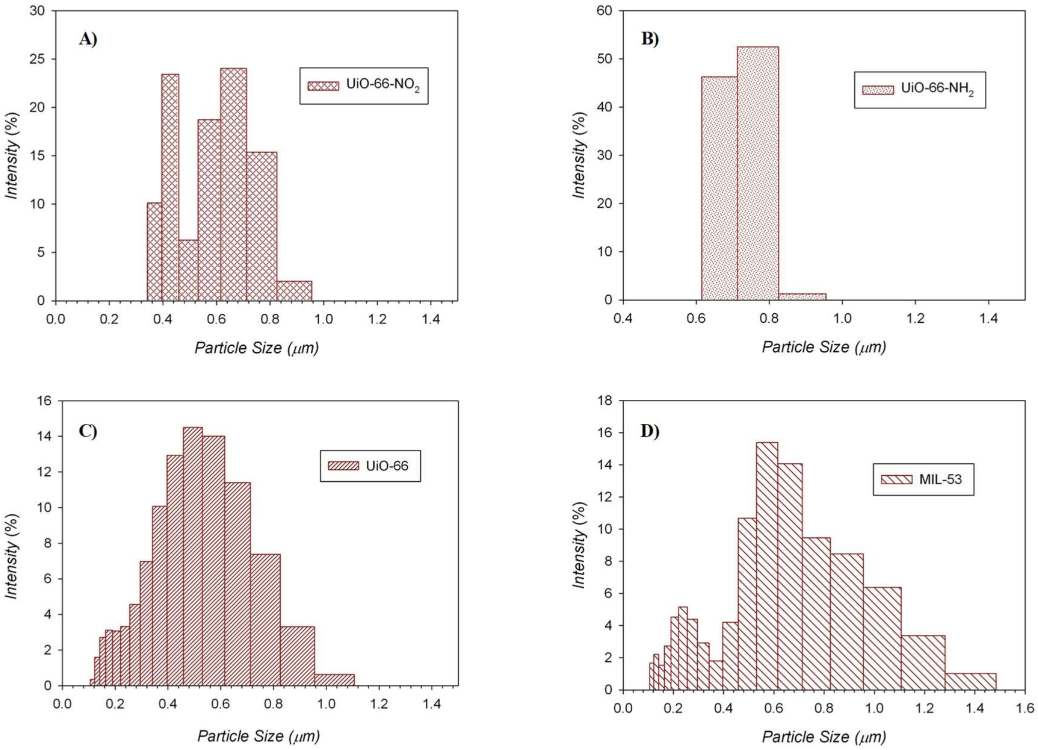

Figure S6. Distribution of the particle sizes for the different MOFs studied: **A**) UiO-66-NO_2; **B**) UiO-66-NH_2; **C**) UiO-66; and **D**) MIL-53(Al).

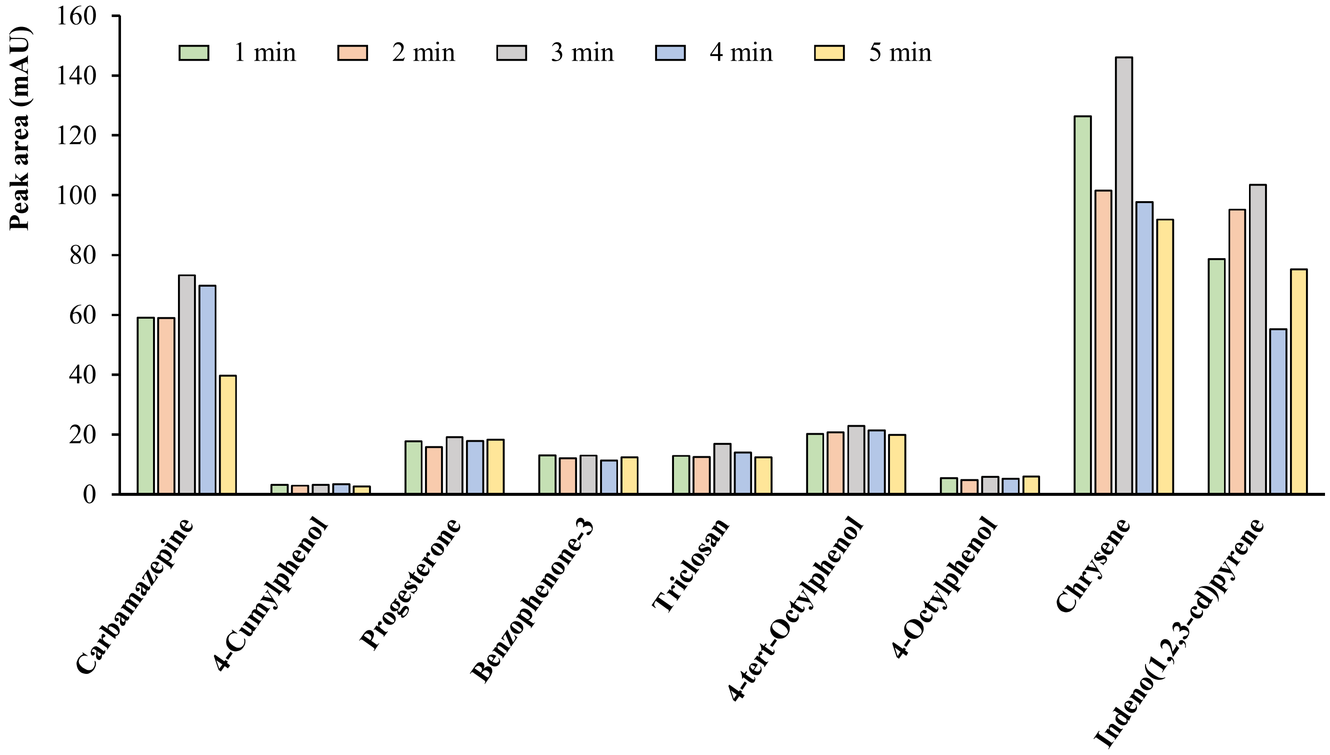

Figure S7. Influence of the extraction time (stirring with vortex) in the extraction efficiency for all analytes in the D-µSPE-HPLC-DAD method. Fixed conditions as described in the text.

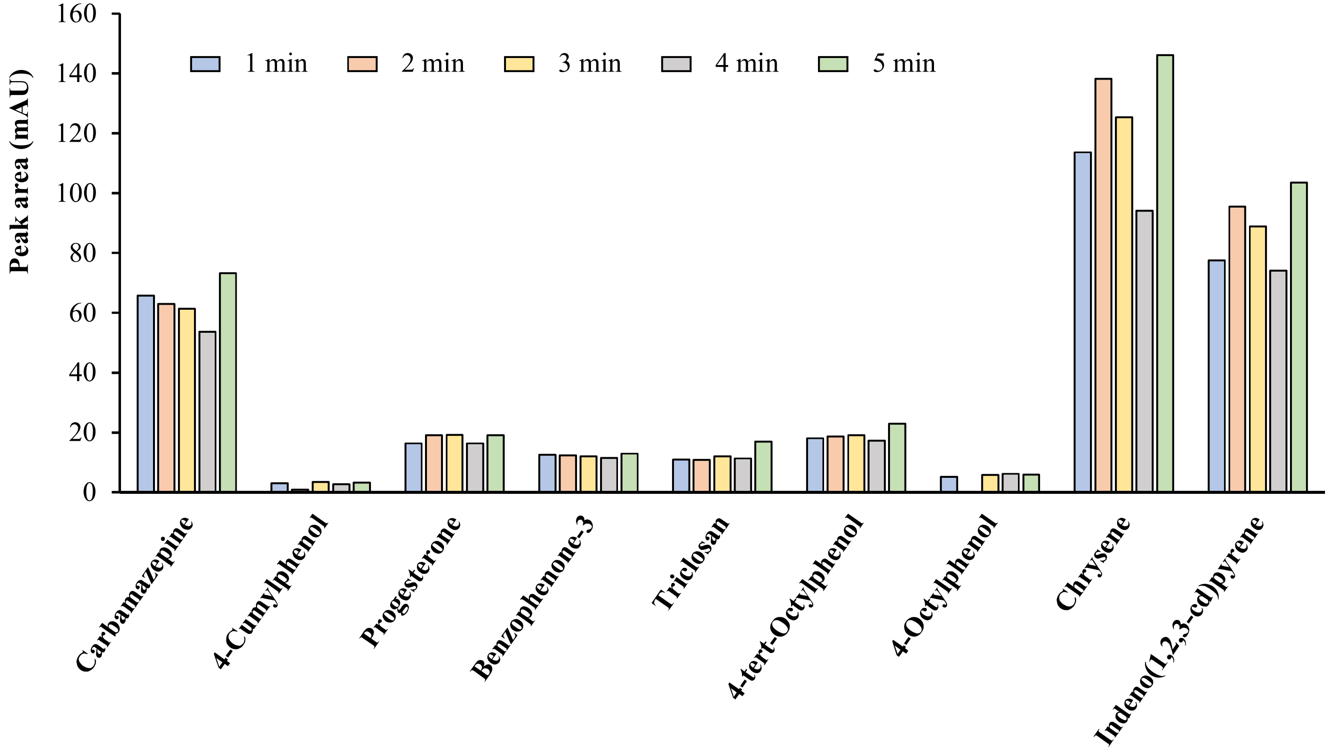

Figure S8. Influence of the elution time (agitation by vortex) in the extraction efficiency of the D-μSPE-HPLC-DAD method. Fixed conditions as described in the text.

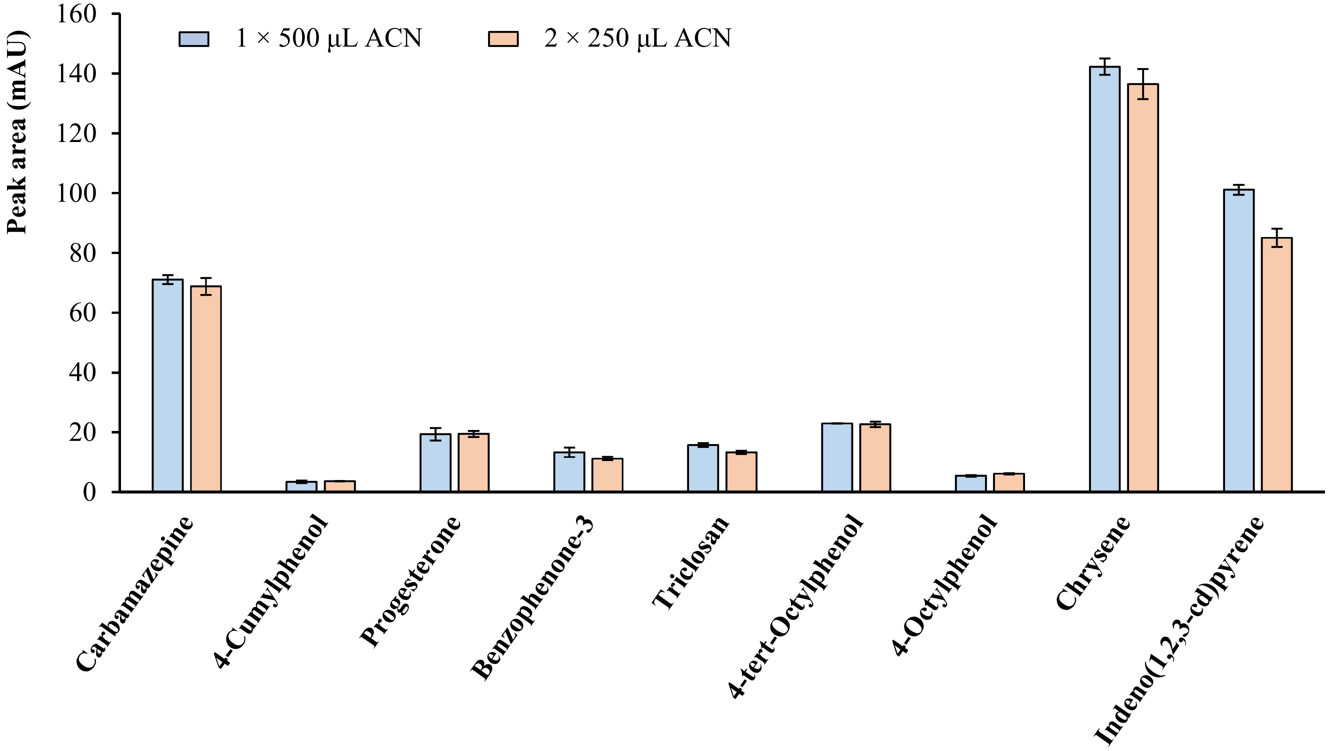

Figure S9. Influence of the number of elution steps in the D-µSPE-HPLC-DAD method using UiO-66-NO_2 as sorbent. Fixed conditions as described in the text. Experiments were carried out in triplicate.

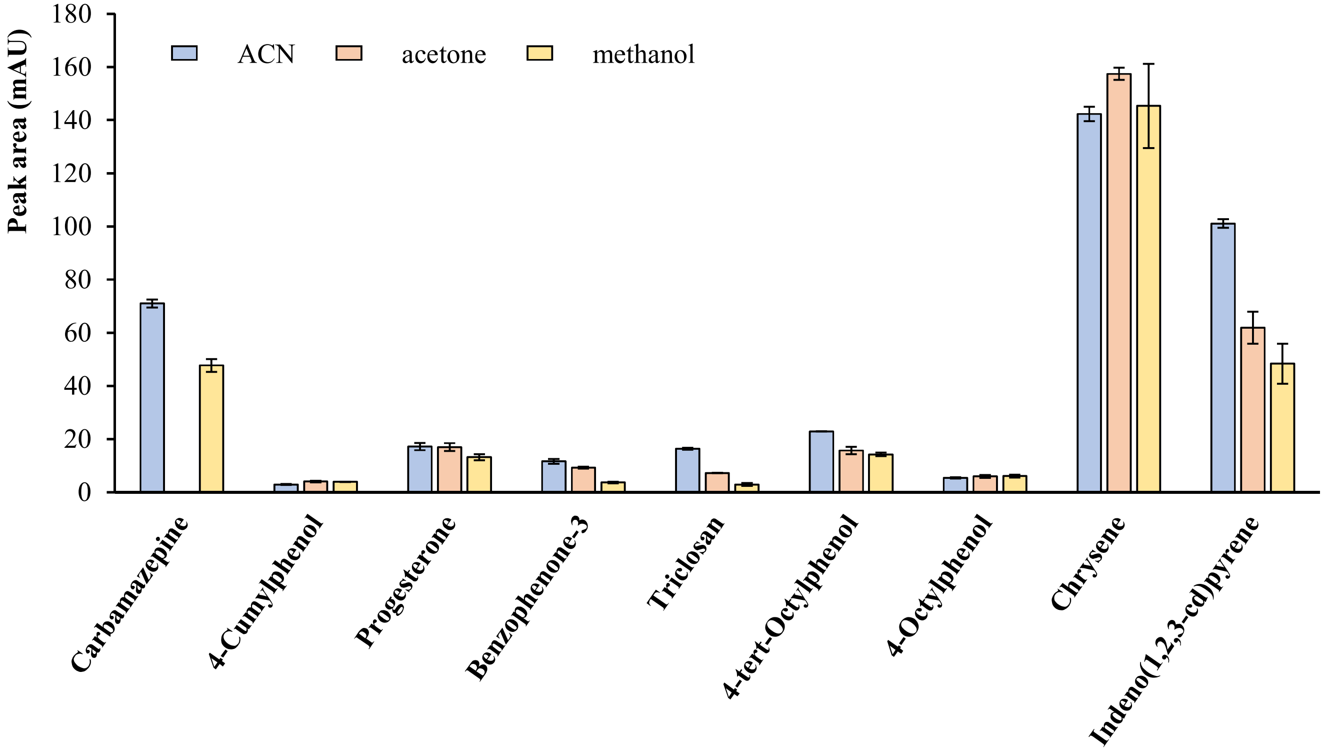

Figure S10. Effect of the nature of the elution solvent in the elution step of the D-µSPE-HPLC-DAD method using UiO-66-NO_2 as sorbent. Fixed conditions as described in the text. Experiments were carried out in triplicate.

Table S1. Structures and several physicochemical properties of the analytes studied.

Analyte (abbreviation)	Structure	Molecular formula<br>Molecular weight[a] (g·mol^{-1})	Molar volume[a,b]<br>(cm^3·mol^{-1})	pKa[a]	Vapor pressure<br>at 25 °C[a] (atm)	Log<br>Kow[a,c]
Carbamazepine<br>(Cbz)	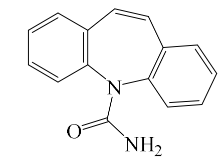	$C_{15}H_{12}N_2O$<br>236.27	186.5 ± 3.0	13.9	$7.61\cdot10^{-10}$	1.90
4-Cumylphenol<br>(CuP)	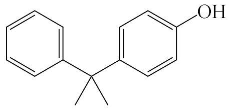	$C_{15}H_{16}O$<br>212.29	201.1 ± 3.0	10.6	$6.55\cdot10^{-8}$	4.24
Progesterone<br>(Pg)	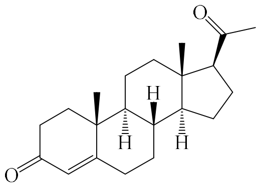	$C_{21}H_{30}O_2$<br>314.46	288.9 ± 5.0	-	$4.53\cdot10^{-11}$	3.83
Benzophenone-3<br>(BP-3)	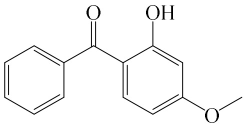	$C_{14}H_{12}O_3$<br>228.24	189.9 ± 3.0	7.6	$6.92\cdot10^{-9}$	4.00
Triclosan<br>(Tr)	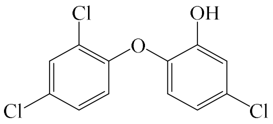	$C_{12}H_7Cl_3O_2$<br>289.54	194.3 ± 3.0	7.8	$4.29\cdot10^{-8}$	5.34

Analyte (abbreviation)	Structure	Molecular formula<br>Molecular weight[a] ($g\cdot mol^{-1}$)	Molar volume[a,b]<br>($cm^3\cdot mol^{-1}$)	pK_a[a]	Vapor pressure at 25 °C[a] (atm)	Log K_{ow}[a,c]
4-*tert*-Octylphenol<br>(*t*-OP)	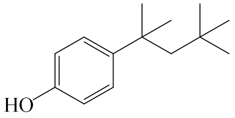	$C_{14}H_{22}O$<br>206.32	220.6 ± 3.0	10.2	$2.61\cdot10^{-6}$	5.18
4-Octylphenol<br>(OP)	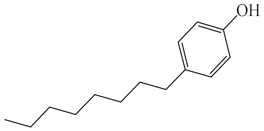	$C_{14}H_{22}O$<br>206.32	219.7 ± 3.0	10.2	$3.29\cdot10^{-7}$	5.63
Chrysene<br>(Chy)	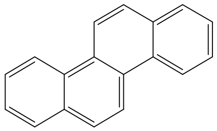	$C_{18}H_{12}$<br>228.29	191.7 ± 3.0	-	$1.12\cdot10^{-10}$	5.73
Indeno(1,2,3-cd)pyrene<br>(Ind)	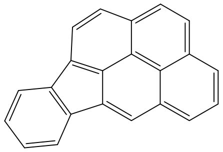	$C_{22}H_{12}$<br>276.33	200.4 ± 3.0	-	$2.05\cdot10^{-12}$	6.65

[a] SciFinder® 2018 database
[b] T = 20 °C; p = 760 torr
[c] *n*-octanol/water partition coefficient

Table S2. Several quality analytical parameters of the HPLC-DAD method.

Analyte	R[a]	$s_{y/x}$[b]	Slope ± SD[c]	LOD[d] (μg·L^{-1})	LOQ[e] (μg·L^{-1})	Calibration range (μg·L^{-1})
Carbamazepine	0.9990	0.80	0.43 ± 0.03	0.50	1.67	2.00 – 100
4-Cumylphenol	0.9992	0.15	0.09 ± 0.01	1.00	3.33	5.00 – 100
Progesterone	0.9990	0.65	0.33 ± 0.02	0.08	0.25	0.30 – 100
Benzophenone-3	0.9992	0.56	0.33 ± 0.01	0.09	0.30	0.50 – 100
Triclosan	0.9983	0.25	0.10 ± 0.01	0.09	0.30	5.00 – 100
4-*tert*-Octylphenol	0.9993	0.15	0.101 ± 0.004	0.50	1.67	5.00 – 100
4-Octylphenol	0.9993	0.12	0.082 ± 0.003	0.50	1.67	3.00 – 100
Chrysene	0.9998	1.9	2.66 ± 0.04	0.02	0.07	0.10 – 100
Indeno(1,2,3-cd)pyrene	0.9997	1.1	1.07 ± 0.02	0.03	0.09	0.10 – 100

[a] correlation coefficient
[b] standard deviation of residuals
[c] confidence intervals for the slope (n = 7) with a signification level of 95%
[d] limit of detection
[e] limit of quantification

Table S3. Intra-day precision study of the HPLC-DAD method.

Analyte	Level 1: 6.0 $\mu g \cdot L^{-1}$	Level 2: 30 $\mu g \cdot L^{-1}$	Level 3: 70 $\mu g \cdot L^{-1}$	RSD (%) retention time[b]	Average retention time (min)
	RSD (%) intra-day[a]	RSD (%) intra-day[a]	RSD (%) intra-day[a]		
Carbamazepine	1.2	2.4	2.2	0.21	1.96
4-Cumylphenol	2.7	2.2	2.2	0.13	6.09
Progesterone	0.9	1.8	1.5	0.12	6.40
Benzophenone-3	2.6	1.3	0.7	0.12	6.62
Triclosan	3.1	3.5	0.9	0.07	7.23
4-*tert*-Octylphenol	3.7	1.5	2.5	0.06	7.63
4-Octylphenol	3.4	2.2	2.9	0.04	9.02
Chrysene	2.3	3.1	1.3	0.08	10.2
Indeno(1,2,3-cd)pyrene	2.8	3.6	1.9	0.12	12.9

[a] relative standard deviation of the calculated concentration using the HPLC-DAD calibration curve (n = 5)
[b] relative standard deviation of the retention times (n = 15)

Table S4. Extraction methods described in the literature for the determination of several of the studied pollutants using solid-based extraction methods and HPLC with UV or DAD detection (to have comparable systems with that of the current study).

Analyte family (number)	Sample prep.	Detector	Sorbent (mg)	Sample (mL)	E_R[a] (%) / spiked level	LOD[b] (µg·L^{-1})	RSD[c] (%)	Ref.
PAHs[d] (4)	D-µSPE[e]	DAD[f]	MNPs[g]-nylon-6 composite (40)	water (25)	79.4 / 20 µg·L^{-1}	0.31	4.2	*J. Chromatogr. A* **2014**, *1345*, 43–49
Benzophenones (3)	D-µSPE[e]	DAD[f]	MIL-101(Cr) (12)	toner (20)	-	0.9	<7.4	*Talanta* **2015**, *132*, 713–718
Drugs (3)	D-µSPE[e]	DAD[f]	Fe_3O_4@polyDA-MWCNTs[h] (15)	plasma (5), CSF[i] (5)	86.5–95.1 / 50–200 µg·L^{-1}	0.4–1.9	<6.4	*Anal. Bioanal. Chem.* **2018**, *410*, 3779–3788
Steroids (3)	MSPE[j]	UV[k]	Fe/CNTs[l]-$SrTiO_3$ (20)	milk (20)	-	0.033	2.31	*Food Anal. Meth.* **2018**, *11*, 3179–3189
Cbz, Pg, Tr (6)	D-µSPE[e]	DAD[f]	MIL-53(Al) (5)	water (10)	32.7 – 61.7 2–8 µg·L^{-1}	0.04–0.15	<8.3	*Talanta* **2018**, *179*, 775–783
Phenols (4)	D-SPE[m]	UV[k]	Fe_3O_4-OA/CQDs[n] (-)	water (20), milk (20)	-	0.09–0.17	<2.8	*J. Mol. Liq.* **2018**, *261*, 155–161
PPCPs[o] (3)	MSPE[j]	UV[k]	Fe_3O_4/GO[p] (20)	water (50)	-	0.63	4.18	*Water Sci. Technol.* **2018**, *77*, 2220–2227

[a] extraction efficiency
[b] limit of detection
[c] relative standard deviation
[d] polycyclic aromatic hydrocarbons
[e] dispersive micro-solid phase extraction
[f] diode array detection
[g] magnetic nanoparticles
[h] multi-walled carbon nanotubes
[i] cerebral spinal fluid
[j] magnetic solid-phase extraction
[k] ultraviolet-visible detection
[l] carbon nanotubes
[m] dispersive solid phase extraction
[n] carbon quantum dots
[o] pharmaceutical & personal care products
[p] graphene oxide

Table S5. Analytical performance of the entire D-µSPE-HPLC-DAD method in terms of relative recovery, extraction efficiency, and inter-day precision with tap water and wastewater samples.

Analyte	Tap water	Wastewater-1	Wastewater-2	Wastewater-1 (spiked level: 1.5 µg·L^{-1})		
				E_R[a] (%)	RR[b] (%)	RSD[c] (%)
Carbamazepine	nd	nd	nd	1.15	52.5	13
4-Cumylphenol	nd	nd	nd	36.8	130	17
Progesterone	nd	nd	nd	5.76	17.6	2.8
Benzophenone-3	nd	nd	nd	36.7	133	1.8
Triclosan	nd	nd	nd	92.2	195	4.5
4-*tert*-Octylphenol	nd	nd	nd	5.99	5.04	5.5
4-Octylphenol	nd	nd	nd	15.3	25.8	3.1
Chrysene	nd	nd	nd	14.6	38.9	5.5
Indeno(1,2,3-cd)pyrene	nd	nd	nd	9.92	34.1	2.8

nd: non-detected

[a] extraction efficiency calculated considering the preconcentration achieved with the microextraction method

[b] relative recovery (calculated with calibration curves in ultrapure water)

[c] relative standard deviation (n = 3, intra-day)
